# Electrochemical Performance of Ba_0.5_Sr_0.5_Co_0.8_Fe_0.2_O_3−δ_ in Symmetric Cells With Sm_0.2_Ce_0.8_O_1.9_ Electrolyte for Nitric Oxide Reduction Reaction

**DOI:** 10.3389/fchem.2019.00947

**Published:** 2020-01-23

**Authors:** Huangang Shi, Guowei Chu, Wenyi Tan, Chao Su

**Affiliations:** ^1^School of Environmental Engineering, Nanjing Institute of Technology, Nanjing, China; ^2^Changzhou Power Supply Branch, State Grid Jiangsu Electric Power Co., Ltd., Changzhou, China; ^3^School of Energy and Power Engineering, Jiangsu University of Science and Technology, Zhenjiang, China; ^4^WA School of Mines: Minerals, Energy and Chemical Engineering, Curtin University, Perth, WA, Australia

**Keywords:** electrochemical reduction, symmetric cell, nitric oxide, Ba_0.5_Sr_0.5_Co_0.8_Fe_0.2_O_3−δ_, single chamber

## Abstract

The emission of nitric oxide from the combustion process of fossil fuels causes air pollution problems. In addition to traditional removal methods, nitric oxide can be removed by the electrochemical reduction method. In this study, Ba_0.5_Sr_0.5_Co_0.8_Fe_0.2_O_3−δ_ powders were synthesized using a solid-state reaction method. Symmetrical cells, with Sm_0.2_Ce_0.8_O_1.9_ as the electrolyte and Ba_0.5_Sr_0.5_Co_0.8_Fe_0.2_O_3−δ_ as the electrodes, were prepared as the electrochemical reactor for nitric oxide reduction. In the process of electrochemical reduction, nitric oxide reduction occurs at the cathode and oxygen evolution occurs at the anode. To study the nitric oxide reduction performance of the electrode, impedances of the symmetrical cell in different atmospheres were analyzed. For the nitric oxide conversion in symmetric cells, two different modes, dual chamber and single chamber, were applied. Results demonstrated that the denitrification performance of the double chamber was better but the single chamber mode had other advantages in its simple structure. Presliminary stability results of the single chamber symmetric cell show that the electrochemical reduction of nitric oxide in symmetric cells with BSCF performed most reliably.

## Introduction

When a fossil fuel burns, it produces pollutants such as sulfur dioxide, carbon dioxide, nitrogen oxides, and particulate matter. There are many types of nitrogen oxide and the most important one is nitric oxide (NO). It is abundant in the exhaust gas of thermal power plants, chemical plants, and internal combustion engines. In order to control the emission of NO, selective catalytic reduction (SCR) methods have been used to reduce NO to non-toxic and harmless N_2_, using NH_3_ as the reducing agent (Li et al., 2011; Wang et al., 2019b). However, there are also some problems in traditional SCR technologies, such as high expense of catalysts, sensitivity to temperature, emission of NH_3_ affecting downstream equipment, and so on (Yang and Pan, 2007; Ko et al., 2019; Wang et al., 2019a). In order to realize the reduction of NO, it is also feasible to apply a current. In the process of electrochemical reduction, oxygen ionic conductors are usually used as electrolytes and electrodes are attached on both sides of the electrolyte (Hansen, 2010). The addition of current provides electrons for the reduction of NO. The reduction reaction of NO occurs at the cathode, which converts NO into N_2_ and forms oxygen ions. Oxygen ions, driven by the current, reach the anode through the electrolyte and participate in the oxidation reaction by forming oxygen. In the electrochemical denitrification process, the current is equivalent to the reductant. The current can be input from an external power source and the reactor is an electrolytic cell (Hibino et al., 2000; Li et al., 2019); the current can also be generated *in situ*. In effect, the reactor is a chemical power source, i.e., fuel cell (Huang and Chou, 2010; Wu et al., 2020).

The idea of electrochemical reduction of NO using solid-state cells was proposed by Pancharatnam et al. (1975). They studied the electrochemical reduction of NO with precious metal electrodes over an yttria-stabilized zirconia (YSZ) electrolyte and analyzed the mechanism of electrochemical denitrification. The results showed that, under the action of applied current, NO is reduced to N_2_ at the cathode side and the oxygen is generated is at the anode side. Hibino et al. studied the electrochemical reduction of NO on Sm_0.2_Ce_0.9_O_1.9_ (SDC) electrolyte surface with a precious metal electrode. They explored the effect of the presence of H_2_O, CO_2_, and O_2_ on NO reduction. The results demonstrated that the presence of CO_2_ does not affect the reduction of NO, while both H_2_O and O_2_ have a greater impact on the reduction of NO. They also pointed out that the transfer of oxygen, generated during NO reduction, has a great influence on the conversion of NO. The faster is the oxygen transfer in cathode production, the faster NO is reduced (Hibino et al., 1996). Wachsman et al. studied the application of perovskite materials in electrochemical denitrification. The results showed that the NO conversion performance of La_0.8_Sr_0.2_MnO_3_ (LSM) was better than a Pt cathode over a YSZ electrolyte (Wachsman et al., 2000). Bredikhin et al. also studied perovskite-based electrodes for the electrochemical reduction of NO. It was also revealed that a composite cathode consisting of La_0.6_Sr_0.4_Fe_0.8_Co_0.2_O_3_ (LSCF) and Gd_0.1_Ce_0.9_O_1.93_ (GDC) had the highest selectivity toward NO reduction when the electrode was made relatively thick (Bredikhin et al., 2002).

The electrochemical reduction process of NO is similar to that of O_2_ in the cathode of a solid oxide fuel cell (SOFC), where the materials for O_2_ reduction may have a similar function to that of NO. Precious metals (Colucci et al., 1985), LSM (Tan et al., 2016), LSCF (Hwang et al., 2005), and other common cathode materials (Song et al., 2006) of SOFCs have been studied for NO reduction in solid electrolyte reactors. Perovskite materials were widely used for oxygen evolution and oxygen reduction reactions (Guo et al., 2019; Zhang et al., 2019). The main reason they can be used is that they are good reductive catalysts. In view of the reaction mechanism of electrochemical denitrification, the oxygen ion conductivity of materials also is important. Ba_0.5_Sr_0.5_Co_0.8_Fe_0.2_O_3−δ_ (BSCF) was first used as an SOFC cathode in 2004, showing excellent performance that received a lot of attention, leading to further research (Shao et al., 2000, 2005; Shao and Haile, 2004). Especially at medium and low temperatures, BSCF exhibits high catalytic activity and high oxygen ion conductivity, which has led to it being more widely used in SOFCs (Zhu et al., 2008), oxygen permeable membranes (Zeng et al., 2007), membrane reactors (Wang et al., 2005; Garcia-Fayos et al., 2018), low temperature catalysis (Xu et al., 2016), and other energy and environment fields (Heidari et al., 2017). At present, there is no report on the performance of BSCF in the NO reduction process. In this paper, BSCF was synthesized using a solid-state method. SDC was used as the electrolyte and a simple symmetrical cell structure was used to study the denitrification performance of a BSCF cathode on the SDC electrolyte material. The stability of the BSCF cathode was tested and the possibility of electrochemical denitrification using BSCF as a cathode was explored.

## Materials and Methods

The BSCF powders in this study were synthesized by a solid-state reaction method. All the raw materials used were obtained in analytical grade from Sinopharm Chemical Reagent Co., Ltd., Shanghai, China. To obtain BSCF, stoichiometric amounts of BaCO_3_, SrCO_3_, Fe_2_O_3_, and Co_2_O_3_ were added into ethanol. The mass ratio of ethanol to solid was 4:1. The mixture was ball-milled in a ball mill (QM-3SP2, Instrument Factory of Nanjing University, China) for 24 h and the speed was controlled at 300 rpm. The resulting slurry was dried at 90°C and then calcined at 500–900°C for 24 h with a heating rate of 5°C min^−1^. BSCF powders were obtained by grinding the oxide after calcination. The SDC powders in this study were synthesized by an EDTA-citrate complexing method. According to the stoichiometric ratio, Sm(NO_3_)_3_ · 6H_2_O, Ce(NO_3_)_3_ · 6H_2_O and deionized water were added to a beaker. The mixed solution was stirred and heated until a clarified solution with 1 mol L^−1^ metal ion concentration was formed. According to the molar quantity of metal ions, EDTA of equal molar quantity and citric acid of twice molar quantity were added. In order to clarify the solution again, a proper amount of ammonia water was added to the beaker to adjust the pH value to approximately neutral. The resulting clarified solution was heated and stirred at 80°C until a gel was formed, then transferred to an oven for heating to form a black precursor. After cooling the precursor, grinding it in a mortar and calcining it in a furnace at a heating rate of 5°C min^−1^ for 5 h at 800°C, the calcined powder was ground in a mortar again to obtain the electrolyte powder. To study the behavior of this powder calcination process, thermogravimetric analysis (TGA) of the precursor in an air atmosphere was conducted, with a heating rate of 10°C min^−1^. The crystal structure of the powders at room temperature was examined by X-ray diffraction (XRD, Rigaku Smartlab) with filtered Cu-Kα radiation at a 2θ range of 20–80° with a 2° min^−1^ scan rate. The morphologies of the BSCF electrode were examined by using a Hitachi S-4800 field-emission scanning electron microscope (FESEM).

The electrochemical reduction performance of NO over a BSCF electrode was studied using symmetric cells. SDC was used as the electrolyte, with the BSCF electrode coating both sides of the electrolyte. SDC electrolyte pellets were prepared by dry pressing and calcination. The SDC powder was weighed (0.4 g) and placed in a circular die. It was dry pressed in an oil press with a pressure of 200 MPa. The green pellets were obtained by removing the mold, then calcining for 5 h at 1,400°C. To protect the electrolyte pellets from deformation or damage, the heating and cooling rates were controlled at 1°C min^−1^. Dense electrolyte pellets were obtained after calcination, with diameters and thicknesses of ~12 and 0.8 mm, respectively. The BSCF electrodes were prepared via spray coating. Slurry with a solid content of about 5% (mass fraction) was prepared by mixing BSCF powder with glycerol, isoacetone, and ethylene glycol in a certain proportion and milling in a ball mill for 2 h. BSCF slurry was sprayed on both sides of the electrolyte pellets while on a hot plate with a temperature of 120°C. The surface of the pellets was covered with a polyimide tape to form disk-shaped electrodes on the both sides, the diameters of which were 8 mm. To form the connections of the BSCF electrodes to both sides of the SDC pellets, the green electrode layers were calcined at 1,000°C for 5 h, and the heating and cooling rate was controlled at 2°C min^−1^. To create the current collector layer, silver paste was painted onto both surfaces of the electrodes, which were then dried in air at 150°C.

**Figure 1:**
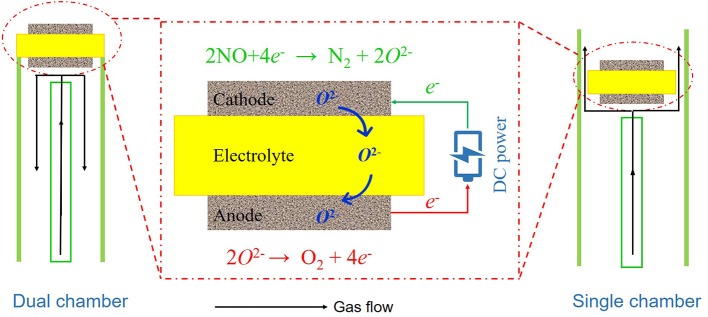
A sketch of nitric oxide (NO) reduction in a symmetric cell with DC power supply.

As shown in Figure 1, we used DC current as the reducing agent for the electrochemical reduction of NO. To investigate the performance of BSCF powders synthesized by a solid-state reaction method, the crystal structure of BSCF powders was tested by XRD. In order to further understand the microstructures of materials and symmetric cells, SEM was used to observe their microstructures. To investigate the performance of BSCF in NO reduction, we tested the AC impedance spectra of symmetric cells at different temperatures using an electrochemical station (VersaSTAT 3, Ametek, USA) at open circuit mode in the frequency range of 100 kHz to 0.1 Hz. AC impedance spectra were recorded at 10 points per decade with an AC perturbation of 10 mV. The symmetric cell was placed in a quartz tube, heated in a tubular furnace, and then fed with the reactive gas. The AC impedance spectra of the cell in different atmospheres were collected. Three kinds of atmosphere were selected, namely pure nitrogen, pure air, and helium containing 1,000 ppm NO.

In order to investigate the NO conversion of symmetrical cells, as shown in Figure 1, we used both dual chamber and single chamber modes to conduct the tests. To calculate the denitrification efficiency, different DC currents were applied on both sides of the cells and the concentrations of NO at the inlet and outlet were record under different temperatures. In this study, NO concentration was recorded via electrochemical sensor with Pt/Pd electrode. Finally, the stability of the symmetric cell as a electrochemical reduction reactor was investigated. The stability time was 5 h, and the concentration of import and export was recorded every 20 min.

## Results and Discussion

**Figure 2:**
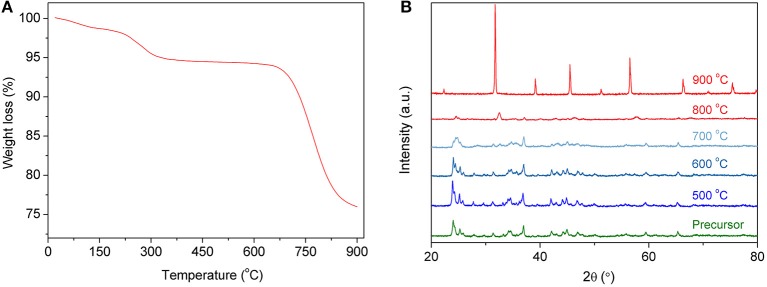
TGA curve of the precursor **(A)** and XRD patterns of the precursor calcined at different temperatures **(B)**.

The solid-state reaction method is a simple method for powder synthesis, which is suitable for industrial preparation of oxide powders, especially for large-scale synthesis. In the process of the solid-state reaction synthesis of powders, it is very important to mix all elements adequately. The BSCF powders reported in this paper were milled in ethanol for 24 h, with corresponding metal carbonates and oxides as raw materials, to achieve full mixing. In the later stage of powder roasting, carbonates, and oxides will be converted into mixed oxides and reassembled into perovskite oxides due to the process in air. Figure 2A shows the thermogravimetric properties of the precursors. As the temperature increases, the material begins to decompose. In particular, the carbonate further decomposes to finally form oxides. As shown in Figure 2A, the carbonate decomposes quickly from about 650°C and becomes stable at 900°C. In order to examine the material structure at different sintering temperatures, XRD analysis was carried out on the powders. Figure 2B shows the XRD spectra of both precursors and calcined powders. It can be seen from the graph that, before calcination, the XRD spectra of the powders show multiple peaks, and there is no fixed crystal structure. After 900°C calcination, the perovskite structure of the powders begins to appear. During the experiment, with the increase of calcination temperature, the agglomeration of powders became more and more obvious, especially after 1,000°C. The agglomeration of powders is very harmful to their later crushing, so 900°C was chosen as the temperature for powder synthesis in this work. When the powder is deposited on the surface of the electrolyte, it will be calcined at a higher temperature for improved adherence to the surface of the electrolyte, further strengthening the crystal structure of the powder and making it a perovskite-structure material.

**Figure 3:**
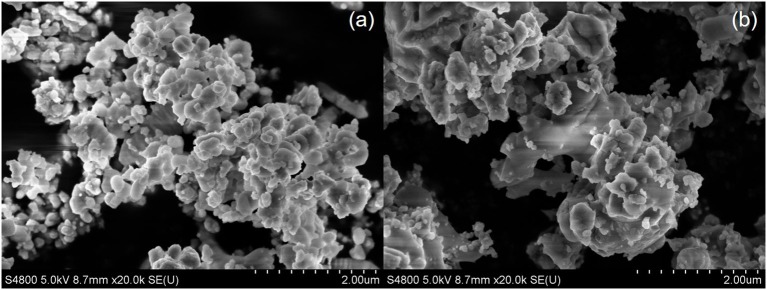
SEM images of the BSCF powder sintered at 600°C **(a)** and 900°C **(b)**.

As shown in Figure 3a, the powder calcined at 600°C for 24 h has the particle size of about 200 nm, with some agglomerations. With the increase in calcination temperature (Figure 3b), the particle size of the powder became larger and more agglomerations were formed.

**Figure 4:**
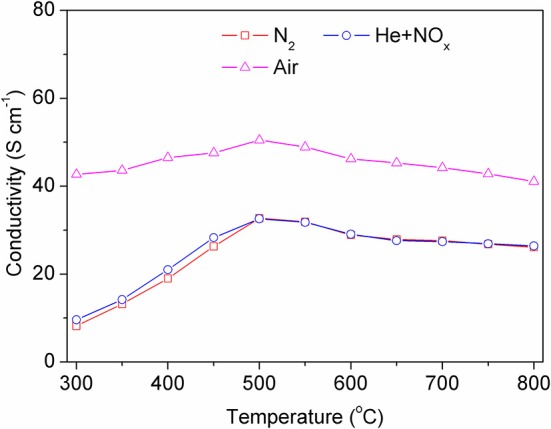
The total conductivity of the BSCF bar in different atmospheres at 300–800°C.

Figure 4 shows the variation of the total conductivity of the BSCF sintering bar in different atmospheres with differing temperatures. The test temperature range is 300–800°C. It can be seen from the figure that the conductivity changes little with temperature, but the conductivity varies greatly under different atmospheres, especially in air and nitrogen atmospheres. BSCF is a typical mixed-conductor material, which has both electronic conductivity and oxygen ion conductivity. When the material is in an oxygen-containing atmosphere, the oxygen ion conductivity will enhance the total conductivity of the material. The conductivity in an NO atmosphere is also low, because the concentration of NO is too low to reflect the contribution of oxygen ion conduction.

**Figure 5:**
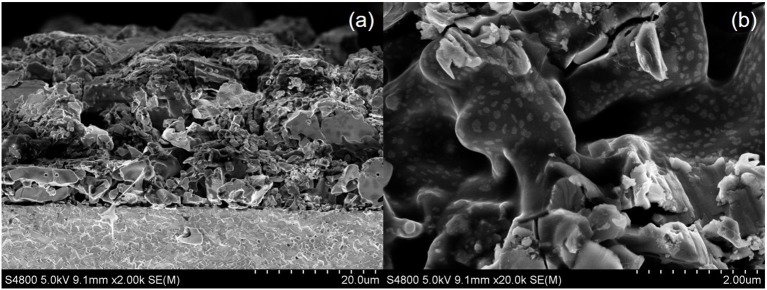
Low magnification **(a)** and high magnification **(b)** cross-sectional SEM images of the cell with BSCF electrode and SDC electrolyte.

In this study, the electrochemical reduction of NO was conducted with a symmetric cell in both single and dual chamber modes. As shown in Figure 5a, the BSCF electrode is porous and the SDC electrolyte is more dense. The BSCF electrode is well-connected to the electrolyte surface and the thickness of the BSCF electrode is about 25 μm. Figure 5b shows the micro morphology of the electrode. It can be seen from Figure 5 that the electrode surface is not fine enough, which is related to the larger particle size of the powder synthesized by the solid-state reaction method.

**Figure 6:**
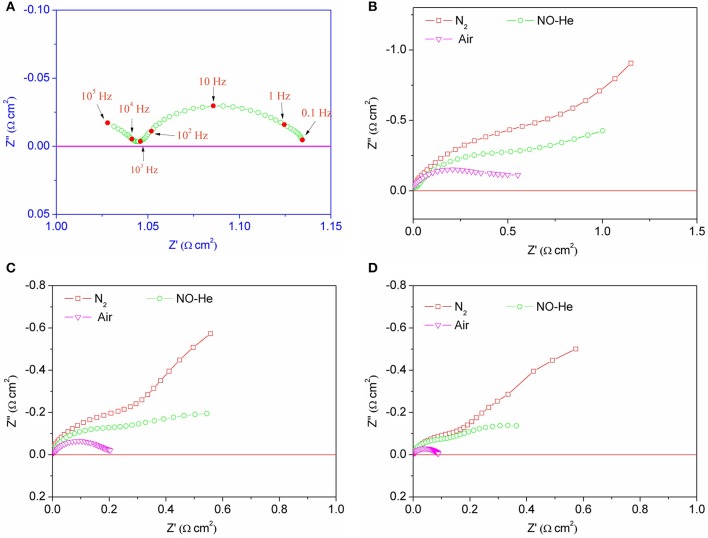
AC impedance spectra of the symmetric cell with BSCF electrodes in air at 700°C **(A)** and in different atmospheres at 600°C **(B)**, 650°C **(C)**, and 700°C **(D)**, respectively.

Figure 6 shows the AC impedance spectra of a symmetrical battery composed of BSCF and SDC in different atmospheres at 600–700°C. Figure 6A displays the AC impedance spectra of the symmetric cell in air at 700°C. Mixed conductor materials have both electronic conductance and oxygen ion conductance. From the comparison of impedance spectra, the changes of impedance values can be clearly observed, especially at low frequencies. The results show that the impedance of the electrode was very small in air and very large in the no-oxygen atmospheres, especially at low frequency. Compared with the nitrogen atmosphere, the impedance of the symmetric cell in the NO-containing atmosphere was of smaller value. This result shows that the reduction of NO occurred at the electrode and provided oxygen ions to reduce the impedance, especially at low frequencies.

**Figure 7:**
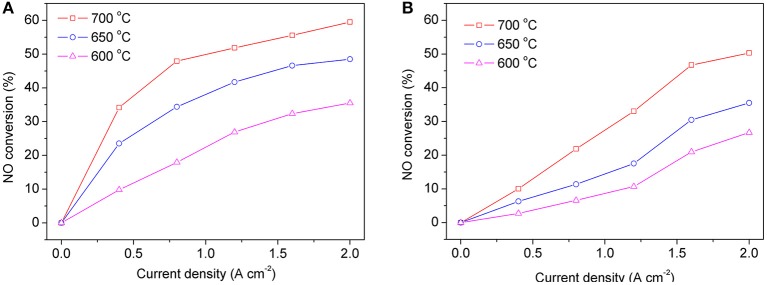
NO conversion at different operating temperatures in dual chamber mode **(A)** and single chamber mode **(B)** of the symmetric cells.

When the symmetrical cell works with two chambers, the gas compositions on each side of the electrode are different. One side of the electrode is a mixture of NO and He, and the other side is air. Figure 7A shows the curves of NO conversion with changing current density when operating in dual chamber mode with operating temperatures of 600–700°C. At each operating temperature, as current density increases, the conversion of NO also increases, however the rate of change in NO conversion gradually lessens. When the current density reaches 2 A cm^−2^, the NO conversion at 600, 650, and 700°C is 33, 43, and 60%, respectively. For comparison, we also tested the denitration performance of the symmetrical cell in the single chamber operation mode. As can be seen in Figure 7B, similar to the double chamber operation mode, NO conversion increases with the increase of both temperature and current density, while the NO conversion also increases with the increase of current density at each individual operation temperature. These results are very closed to work of Moon et al., which was also used single chamber to conducted the NO decomposition (Moon et al., 2002).

**Figure 8:**
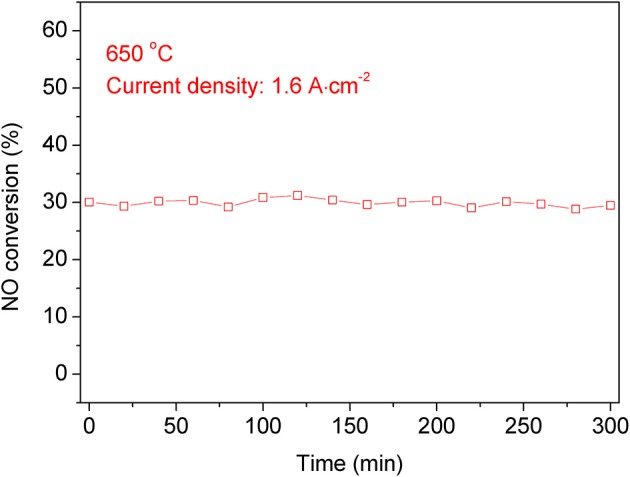
The NO conversion stability of the symmetric cell at 650°C with a current density of 1.6 A cm^−2^ for 5 h.

Compared with the double chamber operation mode, the single chamber operation mode has a simpler structure. If it is used in industry, it can be directly installed in the flue or exhaust pipe. In order to test the stability of NO conversion, we tested the stability of the single chamber operation mode. As shown in Figure 8, when the current density is 1.6 A cm^−2^, the conversion rate of NO is ~33% over a 300 min period. Although there is some fluctuation in the process, the overall conversion rate is still stable.

## Conclusions

As reported in this paper, BSCF powder was synthesized by a solid state reaction method and used for the electrochemical reduction of NO. BSCF powder with a perovskite phase was prepared by mechanical mixing and high temperature calcination at 900°C. The impedance values of symmetrical cells in different atmospheres demonstrated that NO can be reduced on the surface of a BSCF electrode, and provide oxygen ion conductivity for the electrode, reducing its polarization impedance, especially at a low frequency. When utilized as an electrochemical reactor for NO, the double chamber operation mode achieved higher NO conversion than that of the single chamber mode because there was no back mixing between the gases on both sides of the electrode. Although the NO conversion rate of the symmetrical cell was low, the simple configuration of a single cell has some advantages. The 5 h stability test showed that, in a short time, the symmetrical cell can maintain a relatively stable electrochemical denitrification efficiency, so it has a certain potential practical application value.

## Data Availability Statement

All datasets generated for this study are included in the article/supplementary material.

## Author Contributions

HS and GC conducted the experiment. CS contributed conception and design of the manuscript, while HS wrote the first draft. All authors contributed to manuscript revision and approved the submitted version.

### Conflict of Interest

GC was employed by Changzhou Power Supply Branch, State Grid Jiangsu Electric Power Co., Ltd. The remaining authors declare that the research was conducted in the absence of any commercial or financial relationships that could be construed as a potential conflict of interest.
